# Virtual Screening of FDA-Approved Compounds: Exploring New Alternatives for HIV Treatment

**DOI:** 10.1021/acsomega.5c06562

**Published:** 2026-02-18

**Authors:** Daniela P. Martinez, Frederico S. Kremer

**Affiliations:** Technological Development Center, 37902 Federal University of Pelotas, Pelotas, Rio Grande do Sul 96010-610, Brazil

## Abstract

Human immunodeficiency virus (HIV) infection remains a significant public health challenge, particularly because of the emergence of drug-resistant strains against the drugs currently used in highly active antiretroviral therapy (HAART). The ongoing search for new molecules with therapeutic potential remains crucial. In this study, a virtual screening approach was employed to identify novel candidates with therapeutic potential for HIV. High-throughput screening (HTS) data were used to train and validate quantitative structure–activity relationship (QSAR) models, which were subsequently applied to screen a library of Food and Drug Administration (FDA) approved molecules. The most promising compounds were further evaluated through molecular docking assays and pharmacokinetic property predictions. This process led to the identification of a set of molecules with the potential for further investigation, demonstrating the effectiveness of this approach in drug discovery and repurposing.

## Graphical abstract

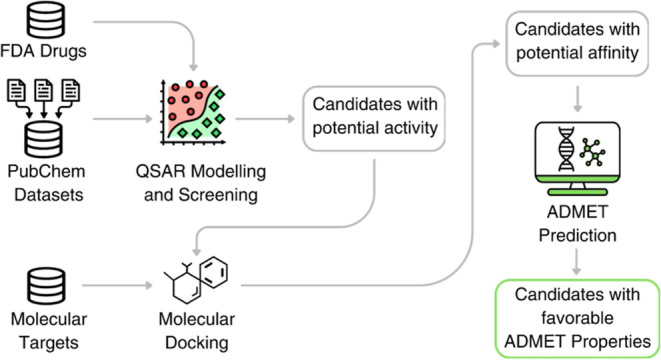

## Introduction

The human immunodeficiency virus (HIV) is a retrovirus that affects approximately 39 million people worldwide. In 2023, approximately 630,000 global deaths were attributed to HIV infection. While still a significant concern, this number represents a 69% reduction in HIV-related deaths since the peak in 2004. This notable decline is primarily attributed to the widespread implementation of highly active antiretroviral therapy (HAART) for infection control. Estimates indicate that 77% of people living with HIV (PLHIV) were receiving HAART in 2023.^1^

HAART’s primary objective is to diminish the morbidity and mortality associated with HIV infection. This objective is achieved by suppressing the viral load to undetectable levels, with viral suppression, PLHIV now experiences a life expectancy and quality of life comparable to those of individuals without the virus.^2^ Moreover, HAART and sustained viral suppression are crucial for reducing the risk of sexual transmission and preventing mother-to-child transmission.^3^ Currently, the concept of U = U (undetectable = untransmittable) exemplifies the profound success of HAART. Individuals with an undetectable viral load pose no risk of HIV transmission to their sexual partners. This understanding has significantly contributed to reducing the stigma associated with HIV and promoting effective treatment adherence among PLHIV.^4^

Currently, over 30 FDA-approved drugs with diverse mechanisms of action are available for HIV treatment. These include nucleoside and nucleotide reverse transcriptase inhibitors (NRTIs), non-nucleoside reverse transcriptase inhibitors (NNRTIs), protease (PIs) and integrase inhibitors (INIs), as well as fusion, attachment, postattachment, and capsid blockers, along with pharmacokinetic enhancers. According to the National Institutes of Health of the United States (NIH-USA), the recommended HAART regimen for treatment-naïve patients typically comprises a combination of two NRTIs alongside a third antiretroviral, wich can be an INI, a PI, or an NNRTI.^5^

Despite these therapeutic advancements, two key challenges in HIV therapy demand ongoing attention: the emergence of drug-resistant strains and the side effects or adverse reactions associated with treatment. These factors frequently lead to poor patient adherence and, consequently, contribute to the development of resistance.^6^ After one year of HAART, researchers estimate that only 50–70% of patients maintain undetectable viral loads. Virological failure remains prevalent, with several associated risk factors.^7^

Among the virus-related factors, the transmission of drug-resistant virus and the emergence of syncytium-inducing viral strains are particularly noteworthy. Therapy-related elements include the limited potency of HAART, poor penetration into sanctuary sites, and drug interactions that impair treatment effectiveness. In clinical care, the inadequate selection of drug combinations and the lack of HAART resistance testing are significant factors. Additionally, patient-related aspects such as poor adherence to therapy, genetic heterogeneity of the CCR5 and CXCR4 coreceptors, variations in the efficacy of intracellular phosphorylation of reverse transcriptase inhibitors, the presence of membrane proteins responsible for protease inhibitors efflux, gradual immune decline, and distinct patterns of HAART absorption and metabolism also influence treatment effectiveness.^7^

Therefore, there is an ongoing need to identify new therapeutic candidates, ideally those that target novel mechanisms, offer improved pharmacokinetic profiles, and exhibit fewer side effects. Traditionally, high-throughput screening (HTS) has been the primary strategy for the discovery and evaluation of new molecules. While highly effective, this methodology is often time-consuming and costly for industry.^8^ Virtual screening, supported by computational methodologies such as the development of quantitative structure–activity relationship (QSAR) models and molecular docking, offers an approach to optimize this process, with several successful cases of chemoinformatics-driven drugs already reported.^9–11^ Furthermore, computational strategies are also useful in predicting pharmacokinetic properties and drug-likeness, which are important parameters to assess in the early stages of new drug development.^12^

Drug repositioning, a strategic approach in drug discovery, leverages virtual screening to identify new therapeutic uses for already approved molecules. This strategy is rooted in two key concepts: the idea that different diseases often share common biological targets, and the principle of pleiotropic drugs. Pleiotropic drugs are compounds that possess multiple therapeutic activities for multiple targets within a single molecule.^13,14^ A significant advantage of drug repositioning is the streamlined regulatory process for market entry, as it already considers primary data on safety and toxicity. This effectively shortens the early development phases and leads to lower overall costs in bringing a new therapy to the market.

Given the possibility that different antiretrovirals may become ineffective in treating HIV over time, it is evident that exploring alternative approaches for developing active molecules against the virus is crucial. This includes both the prospecting of novel compounds and the repositioning of known molecules. The use of computational tools emerges as a promising solution to accelerate the discovery process, optimizing the identification of candidates with therapeutic potential while simultaneously reducing the costs and time associated with drug development. These strategies expand the range of treatment options available, which could positively impact on the quality of life of PLHIV.

## Results

The best selected models, generated from PubChem bioassays, are summarized in Table 1. Models based on different algorithms, including decision trees, logistic regression, and gradient boosting—among others, were selected. The F1 scores ranged from 66.11% to 90.18%, while accuracy values ranged from 56.16% to 90.56%. All metrics were calculated using a balanced data set. The generated QSAR models were used to develop software called BAMBU-HIV, available at http://200.132.101.156:8085/.

**Table 1:** Validation Results of the Best Models Generated from the Protein Inhibition Data^a^

PubChem AID	descriptors	algorithm	accuracy	recall	precision	ROC_AUC	F1
651571	descriptors	decision tree	56.16	86.84	55	53.5	67.35
565	Mol2vec	logistic regression	73.76	73.97	73.97	79.45	73.97
704	Morgan-2048	random forest	87.79	90.45	86.54	94.38	88.45
1117361	Mol2vec	logistic regression	75.89	72.65	77.14	81.01	74.83
624416	Morgan-2048	gradient boosting	82.67	84.25	81.27	90.31	82.73
651604	Mol2vec	logistic regression	69.44	75	71.43	75	73.17
624170	Mol2vec	logistic regression	80.81	75.68	86.15	85.83	80.58
1053197	Morgan-1024	random forest	67.92	61.26	71.81	72.46	66.12
1117319	Morgan-1024	random forest	74.63	66.49	80	79.3	72.62
743269	Morgan-2048	random forest	74.85	67.24	78.87	80.26	72.59
1986	Morgan-2048	extra tree	90.56	90.61	89.77	95.4	90.19
1053136	Morgan-1024	extra tree	80.06	82.02	78.08	86.92	80
463187	Morgan-2048	random forest	83.69	77.3	90.18	90.72	83.24
372	Morgan-2048	extra-tree	76.88	69.39	82.42	82.83	75.34

a ROC_AUC—receiver operating characteristic–area under the curve.

Among the best models, those with an accuracy exceeding 80% were selected for the subsequent stages. Of the 14 studies collected from PubChem, six exhibited an accuracy equal to or greater than 80%. Additionally, the results of the model generated for reverse transcriptase (PubChem AID: 565—accuracy: 73.76%) were included due to the critical role of this target in the HIV viral replication process. Thus, in addition to reverse transcriptase, the best models developed for the Rev protein, glycoprotein gp160, GLS protein, glycoprotein gp41, integrase, and Nef protein were also utilized.

Using the models’ predictions, virtual screening was performed on a library of FDA-approved molecules. Based on the biological activity predictions performed by the QSAR models, the top 100 molecules from the library were selected for molecular docking, ranked according to their average QSAR prediction scores. This criterion was employed to prioritize molecules with a higher likelihood of exhibiting multitarget activity. The complete list of molecules is presented in Table SA of the Supporting Information.

To rank the molecular docking results, the top 20 molecules were selected based on their average docking energy. In the molecular docking assay, only molecules that demonstrated protein–ligand interactions with binding energies (Δ*G*) ≤ −7.0 kcal/mol and relevant residues at the interface were considered. The complete list of molecular docking results is available in the Supporting Information, Table SB. The high-affinity targets most frequently identified among the results were protease (PDB: 5IVQ) and reverse transcriptase (PDB: 3LP0). The molecule pioglitazone (ZINC000000968326) exhibited the highest number of interactions with various targets in the library, interacting with five distinct targets (PDB: 3F9K, 3LP0, 5IVQ, 5UQE, 6B72). The binding energy ranged from −7.4 to −11.6 kcal/mol, the binding efficiency from −0.30 to −0.40, and the average PLIF score from 30.67% to 34.84%.

Table 2 presents the toxicological profiles of the molecules analyzed using the ADMETLab 3.0 tool. This tool provides values ranging from 0 to 1, with low-toxicity compounds defined as those scoring between 0 and 0.3. Compounds with scores between 0.3 and 0.7 are considered to exhibit medium toxicity, while those above 0.7 are classified as highly toxic. In the neurotoxicity assessment, 10 molecules (10/20) were classified as highly toxic, nine (9/20) as low toxicity, and one (1/20) as medium toxicity. For ototoxicity, most molecules (13/20) exhibited high toxicity, and a similar trend was observed for nephrotoxicity (16/20), genotoxicity (15/20), hepatotoxicity (15/20), and respiratory toxicity (14/20).

**Table 2:**
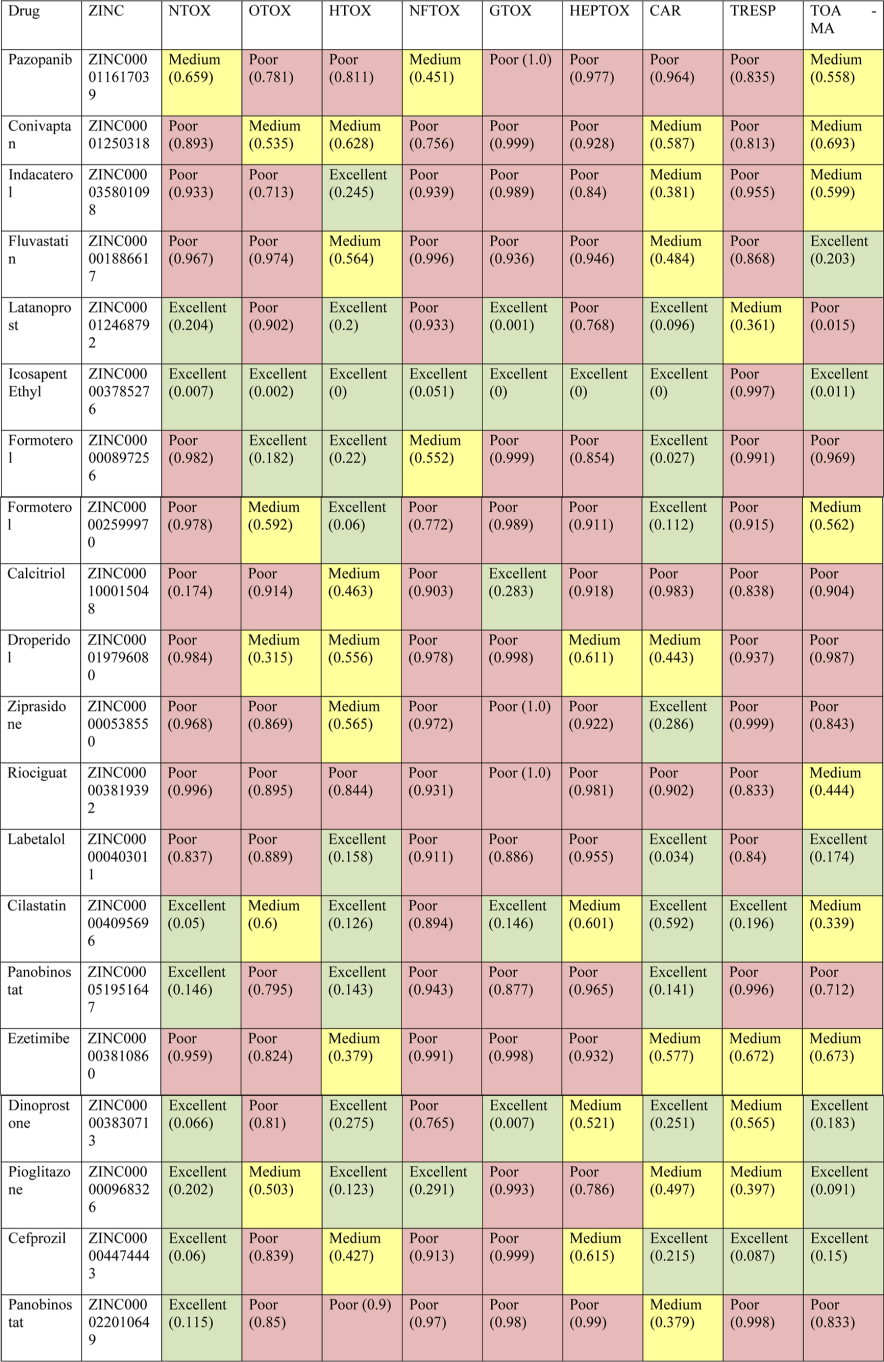
Toxicological Analysis Profile of the Molecules from the FDA-Approved Compound Library, Performed Using the ADMETLab 3.0 Tool^a^

a NTOX = neurotoxicity, OTOX = ototoxicity, HTOX = hematotoxicity, NFTOX = nephrotoxicity, GTOX = genotoxicity, HEPTOX = hepatotoxicity, CAR = carcinogenicity, TRESP = respiratory toxicity, TOA-MA = acute oral toxicity in animal model.

Hematotoxicity was classified as low in 10 molecules (10/20), medium in seven (7/20), and high in three (3/20). Regarding carcinogenicity, 10 molecules were evaluated as having low carcinogenicity (10/20), seven as medium (7/20), and three (3/20) as high. Finally, in the evaluation of acute oral toxicity in an animal model, seven molecules (7/20) were considered low toxicity, another seven as medium (7/20), and six (6/20) as high toxicity. Icosapent ethyl (ZINC000003785276) exhibited the most favorable profile, presenting low toxicity in eight of the nine parameters evaluated and being classified as highly toxic only for respiratory toxicity. In contrast, riociguat (ZINC000003819392) was deemed highly toxic in eight of the nine parameters, with only acute oral toxicity in an animal model classified as medium.

Table 3 presents the drug-likeness profile of the 20 molecules evaluated using the ADMETLab 3.0 tool. Lipinski’s rule stipulates that the molecular mass must be ≤500, the log *P* (octanol/water partition coefficient) must be ≤5, the number of hydrogen bond acceptors (Hacc) must be ≤10, and the number of hydrogen bond donors (Hdon) must be ≤5. According to the rule, for a drug to possess a favorable oral activity profile, it should not violate more than one of these parameters.^15^ Molecules that comply with Lipinski’s rule are classified as “excellent,” while those exhibiting two or more violations of the expected parameters are categorized as “poor.” Notably, all 20 evaluated molecules met the requirements of Lipinski’s rule (20/20).

**Table 3:** Drug-likeness Profile of the Top 20 Molecules from the FDA-Approved Compound Library, Evaluated Using the ADMETLab 3.0 Tool

drug	zinc ID	Lipinski’s rule	Pfizer’s rule	GSK’s rule
pazopanib	ZINC000011617039	excellent	excellent	**poor**
conivaptan	ZINC000012503187	excellent	excellent	**poor**
indacaterol	ZINC000035801098	excellent	excellent	excellent
latanoprost	ZINC000012468792	excellent	excellent	**poor**
icosapent ethyl	ZINC000003785276	excellent	**poor**	**poor**
formoterol	ZINC000000897256	excellent	excellent	excellent
formoterol	ZINC000002599970	excellent	excellent	excellent
calcitriol	ZINC000100015048	excellent	**poor**	**poor**
droperidol	ZINC000019796080	excellent	**poor**	excellent
ziprasidone	ZINC000000538550	excellent	**poor**	**poor**
riociguat	ZINC000003819392	excellent	excellent	**poor**
labetalol	ZINC000000403011	excellent	excellent	excellent
cilastatin	ZINC000004095696	excellent	excellent	excellent
panobinostat	ZINC000051951647	excellent	excellent	excellent
ezetimibe	ZINC000003810860	excellent	**poor**	**poor**
dinoprostone	ZINC000003830713	excellent	excellent	excellent
pioglitazone	ZINC000000968326	excellent	excellent	excellent
cefprozil	ZINC000004474443	excellent	excellent	excellent
panobinostat	ZINC000022010649	excellent	excellent	excellent

According to the Pfizer rule, compounds with log *P* > 3 and a topological polar surface area (TPSA) < 75 are considered potentially toxic. Of the evaluated molecules, five (5/20) met both criteria and were classified as *poor*, while 15 molecules (15/20) met none or only one parameter, and were thus classified as *excellent*.^16^ The GSK rule stipulates that for compounds to exhibit a favorable ADMET profile, the molecular mass must be ≤400 and the log *P* ≤ 4.^17^ According to this rule, 11 (11/20) of the evaluated molecules were classified as *excellent* for meeting both criteria. Conversely, nine (9/20) did not satisfy at least one parameter and were classified as *poor*. Overall, ten molecules (10/20) were considered excellent across all rules utilized to assess drug-likeness in the tool. None of the top 20 molecules in the ranking list of the FDA-approved compound library exhibited PAINS-type interference.

## Discussion and Conclusion

The use of ligand- and receptor-based virtual screening approaches enabled the identification of 20 FDA-approved molecules with potential for repurposing or repositioning. These molecules displayed affinity for various targets, and in some cases, multiple targets of interest for HIV.

### Oncology Drugs

Pazopanib (ZINC000011617039) is an approved antineoplastic multikinase inhibitor used in renal cell carcinoma and soft tissue sarcoma, targeting VEGFR-1, -2, and -3, PDGFR-α/β, and FGFR-1 and -3, thereby inhibiting tumor angiogenesis.^18,19^ In this study, pazopanib exhibited potential interaction with HIV protease (PLIF 35.28%). A retrospective analysis in PLHIV receiving oncological treatment reported that concomitant use of HAART with raltegravir and multikinase inhibitors did not compromise viral suppression, with undetectable viral loads and adequate plasma concentrations of the kinase inhibitors, suggesting a favorable safety profile for this combination.^20^

To date, kinase inhibitors have primarily been employed in the treatment of cancer and inflammatory conditions. However, kinases are known to play significant roles in various physiological processes, making them promising pharmacological targets for further investigation. García-Cárceles reviewed the potential of kinase inhibitors as antiviral agents, emphasizing the connection between protein kinase inhibition and antiviral activity. Notably, they highlight dasatinib, an inhibitor that can suppress the activity of Src proteins, which are crucial for T cell activation, thereby creating an environment less conducive to HIV replication.^21^

Despite these findings, pazopanib exhibited a high predicted toxicity profile in this study, consistent with its known risk of hepatotoxicity, which is highlighted in its clinical labeling.^22^ While current clinical practice recognizes that the benefits of HAART outweigh hepatotoxicity risks^23^ additional studies are required to further assess the safety and therapeutic feasibility of pazopanib in the context of HIV treatment.

Panobinostat (ZINC000051951647 and ZINC000022010649) is a nonselective histone deacetylase inhibitor approved for the treatment of multiple myeloma, acting through epigenetic modulation of gene expression and inhibition of protein metabolism.^24^ In the molecular docking assays, this drug demonstrated multiple potential interactions with reverse transcriptase, protease, and integrase (PLIF scores of 31.20% and 30.67%). Additionally, QSAR models suggested a possible interaction with gp41, although this was not detected in docking analyses.

When administered in conjunction with interferon-α2a, panobinostat appears to induce structural transformations in HIV-1 reservoir cells. The pharmacological induction of proviral gene expression may facilitate immunological clearance, aligning with the principles of “shock and kill” therapies designed to reduce HIV reservoirs. Armani-Tourret and colleagues demonstrated that the combined administration of panobinostat and interferon resulted in a decrease in nonclonal intact proviruses, a reduction in the structural and phylogenetic complexity of intact proviruses, and an increase in the number of proviruses with 5-LTR deletions. While such effects may occur naturally during long-term HAART, they appear to be accelerated by the described protocol, indicating that HIV-1 proviruses may be rendered and sensitized for immunological clearance.^25^ These findings are supported by clinical evidence demonstrating significant latency-reversing activity in PLHIV treated with panobinostat.^26^

Additionally, the drug has been associated with reduced levels of plasma markers of inflammation, including C-reactive protein, interleukin-6 (IL-6), matrix metalloproteinase 9, and E-selectin in PLHIV. The suppression of these markers suggests a potential therapeutic benefit of panobinostat in PLHIV, particularly in individuals at high cardiovascular risk.^27^ Nevertheless, panobinostat exhibits a high toxicity profile, a common feature of oncological agents, underscoring the need for rigorous evaluation of the risk–benefit balance when considering its repurposing for HIV-related therapeutic strategies.

### Medications for Pulmonary Conditions

Indacaterol (ZINC000035801098) and formoterol (ZINC000000897256 and ZINC000002599970) are long-acting β_2_-adrenergic agonists used for the management of asthma and chronic obstructive pulmonary disease by promoting bronchial muscle relaxation. Formoterol presents a comparatively more favorable toxicity profile, although both agents share common adverse effects such as nasopharyngitis and headache.^28^

In this study, indacaterol (PLIF 34%) and formoterol (PLIF 32.13% and 32%) exhibited potential interactions with HIV reverse transcriptase and protease. QSAR models predicted high probabilities of interaction between indacaterol and the GLS protein (97.88%) and gp120 (72.91%), as well as between formoterol and GLS (97.96% and 96.53%), although these interactions were not supported by molecular docking analyses.

Evidence from the literature suggests potential antiviral activity of formoterol beyond its bronchodilator effects. In combination with budesonide, formoterol has been shown to reduce virus-induced inflammation by modulating pro-inflammatory cytokines and the type I interferon pathway, particularly in rhinovirus infections.^29^ Similar immunomodulatory effects have been reported in pediatric viral pneumonia,^30^ and drug repositioning studies have identified formoterol as a potential inhibitor of enterovirus A71 replication during early infection stages.^31^

Riociguat (ZINC000003819392), a guanylate cyclase stimulator, is approved for the treatment of thromboembolic pulmonary hypertension and pulmonary arterial hypertension. The latter is a serious complication associated with HIV infection. DeJesus evaluated pharmacokinetic interactions of riociguat in patients undergoing HAART and reported an increase in the ROC curve of riociguat exposure in patients receiving the antiretroviral regimen abacavir/dolutegravir/lamivudine. Despite this pharmacokinetic alteration, the authors emphasized the overall safety of using riociguat in this patient population.^32^

In this study, riociguat showed potential interaction with the GLS protein, which may be relevant in the context of HIV-associated dementia. However, the compound exhibited a high predicted toxicity profile, being classified as high risk in most evaluated parameters, and remains contraindicated during pregnancy due to teratogenic risk.^33^ These findings underscore the importance of a careful risk–benefit assessment before considering riociguat for broader therapeutic applications. To date, there are no reports describing interactions between riociguat and HIV molecular targets or its potential role in HIV-associated dementia, indicating that this remains an unexplored area that warrants further investigation.

### Medications for Cardiovascular Conditions

Fluvastatin (ZINC000001886617) is a hydroxymethylglutaryl-coenzyme A reductase inhibitor widely used to reduce plasma cholesterol levels. Although generally well tolerated, fluvastatin is associated with hepatotoxicity, which is consistent with the toxicity profile predicted in this study. In molecular docking analyses, fluvastatin demonstrated potential interaction with HIV reverse transcriptase (PLIF 33.83%).

The addition of fluvastatin to the therapeutic regimen of patients coinfected with HIV and HCV has been associated with improved virological response.^34^ Moreover, statins exhibit immunomodulatory and anti-inflammatory properties, which are particularly relevant in the PLHIV population, characterized by chronic immune activation. Patients receiving HAART in combination with statins have shown reduced levels of inflammatory markers such as IP-10, IL-10, and IL-12p70.^35^

Fluvastatin has also been reported to interfere with the replication of multiple enveloped and nonenveloped viruses, and other statins, such as lovastatin, have demonstrated the ability to reduce HIV viral load by disrupting host-cell signaling pathways involved in viral entry.^36,37^ Additionally, statin use has been associated with decreased expression of host proteins involved in viral replication in SARS-CoV-2 infection.^38^ These antiviral effects are thought to be mediated, at least in part, by alterations in membrane lipid rafts, limiting cholesterol availability required for efficient viral fusion and gp41-mediated entry.^39^

Ezetimibe (ZINC000003810860), indicated for primary and mixed hyperlipidemia, acts by inhibiting the sterol transporter NPC1L1 without interfering with the absorption of fat-soluble nutrients.^40^ Based on the results obtained in this study, the toxicity profile of ezetimibe can be classified as medium to high. Ezetimibe demonstrated potential interactions with reverse transcriptase and protease (PLIF 31.11%). Although QSAR models predicted potential interactions with integrase (78.14%) and the GLS protein (97.82%), these were not confirmed through molecular docking. Preliminary studies have suggested an antiviral effect of ezetimibe against various viruses, including hepatitis B virus^41^ hepatitis C virus,^42^ as well as dengue, zika, and yellow fever virus.^43^

Icosapent ethyl (ZINC000003785276), an ethyl ester of eicosapentaenoic acid, is approved for the treatment of severe hypertriglyceridemia and is characterized by a favorable safety profile.^44^ Among the compounds evaluated, it showed the lowest predicted toxicity and exhibited potential interactions with HIV integrase and reverse transcriptase in docking analyses (PLIF 32.43%), and a strong probability of interaction with integrase was predicted in a model derived from bioassay 1053136 (99.99%). To date, there are no documented interactions in the literature between ezetimibe and icosapent ethyl with the targets identified in this study.

Despite its safety profile, the immunomodulatory effects of polyunsaturated fatty acids such as icosapent ethyl warrant caution. While supplementation may be beneficial in infections marked by excessive inflammation, prolonged use can alter gut microbiota and dampen immune responses, potentially compromising host defense in certain infectious contexts.^45^

Labetalol (ZINC000000403011), is an α- and β-adrenergic antagonist approved for the treatment of hypertension and related cardiovascular conditions, acting primarily through vasodilation.^46^ In this study, labetalol exhibited potential multitarget interactions with HIV reverse transcriptase, protease, GLS protein, and Nef protein (PLIF 31.56%). Although QSAR models predicted a strong interaction with the GLS protein, a high-probability interaction with integrase was not supported by molecular docking results. Despite the probable multitarget interactions identified in this study, there are no prior reports in the literature documenting labetalol’s interactions with the evaluated receptors. Its toxicity profile, as determined in this study, is considered high, particularly regarding nephrotoxicity and hepatotoxicity.

### Medications from Diverse Classes

Conivaptan (ZINC00001250318) is a vasopressin receptor isotype inhibitor indicated for the treatment of hyponatremia.^47^ In molecular docking assays, it demonstrated a potential interaction with protease (PLIF 34.84%). Although QSAR models suggested a possible interaction with reverse transcriptase, this finding was not supported by docking analyses. Notably, virtual screening studies have identified conivaptan as a potential antiviral candidate against mpox virus and SARS-CoV-2, supporting its broader antiviral relevance.^48,49^

Latanoprost (ZINC000012468792) is a prostaglandin F2α analogue prodrug widely used for the treatment of elevated intraocular pressure, with minimal systemic adverse effects.^50^ In this study, latanoprost demonstrated potential interaction with HIV reverse transcriptase (PLIF 32.47%). While QSAR models predicted a high probability of interaction with integrase, this result was not confirmed by molecular docking.

Calcitriol (ZINC000100015048), the active metabolite of vitamin D, is widely used in the management of secondary hyperparathyroidism and psoriasis.^51,52^ In molecular docking assays, calcitriol exhibited a potential interaction with protease (PLIF 31.91%). The QSAR model suggested a possible interaction with integrase (99.85%), which was not confirmed through docking. Evidence suggests that vitamin D and its metabolites may exert beneficial effects in PLHIV through immunomodulatory mechanisms. Vitamin D has been associated with reduced HIV-1 infection in T cells, decreased chronic inflammation, and improved immunological outcomes, including increased CD4 T cell counts and reduced viral load, supporting its potential role as an adjunct to HAART.^51,53,54^

Droperidol (ZINC000019796080) is a dopamine antagonist derived from butyrophenone, used to prevent and treat nausea and vomiting in postsurgical patients.^55^ In molecular docking assays, it exhibited multiple potential interactions with integrase, reverse transcriptase, protease, and the GLS protein (PLIF 31.89%).

Ziprasidone (ZINC000000538550) is an atypical antipsychotic used in the treatment of schizophrenia and bipolar disorder and is also prescribed for HIV/AIDS-related psychosis.^56^ As a CYP3A4 substrate, its plasma concentrations may increase when coadministered with protease inhibitors such as ritonavir, and its potential to prolong the QT interval requires caution, particularly in combinations with other QT-prolonging agents.^57^ In molecular docking assays, ziprasidone demonstrated potential interactions with HIV protease and the GLS protein (PLIF 31.76%).

Cilastatin (ZINC000004095696) demonstrated potential interactions with protease and reverse transcriptase (PLIF 31.34%). QSAR models predicted possible interactions with integrase and the GLS protein, which were not confirmed in molecular docking assays. Cilastatin functions as an inhibitor of renal dehydropeptidase, an enzyme responsible for metabolizing β-lactam antibiotics. It is clinically used in combination with imipenem to prevent the metabolism of the antibiotic.^58^

Dinoprostone (ZINC000003830713), a naturally occurring prostaglandin E_2_, is clinically used for labor induction and related obstetric indications.^59^ In this study, dinoprostone demonstrated potential interaction with HIV reverse transcriptase in molecular docking assays (PLIF 31.06%). Although QSAR models predicted interactions with integrase, GLS protein, gp160, and gp120, these were not confirmed by docking. Notably, prostaglandin E_2_ has been implicated in HIV replication dynamics, and postinfection exposure has been shown to reduce viral replication by impairing the intracellular transport and assembly of viral components, thereby decreasing virion release.^60^

Pioglitazone (ZINC000000968326) exhibited potential multitarget interactions with integrase, reverse transcriptase, protease, GLS protein, and Nef protein in molecular docking assays (PLIF 30.87%). Notably, none of these interactions were predicted with high probability (>70%) by QSAR models. Originally developed as an antihyperglycemic agent for type 2 diabetes mellitus, pioglitazone has fallen into disuse over the years due to multiple adverse effects, including congestive heart failure and bladder cancer. Its use in PLHIV is particularly associated with metabolic-associated fatty liver disease, a complication in this population that can progress to cirrhosis.^61^

In a study with 98 patients receiving HAART and pioglitazone for 48 weeks, no serious adverse effects were reported, and the drug significantly improved parameters associated with fatty liver disease.^62^ Additionally, pioglitazone has shown broad in vitro antiviral activity against SARS-CoV-2 variants when combined with pamapimod.^63^

Finally, cefprozil (ZINC000004474443), a cephalosporin-class antibiotic, showed a potential interaction with protease (PLIF 30.86%).^64^ QSAR models predicted additional high-probability interactions with integrase and GLS protein; however, they were not confirmed by molecular docking. Among the drugs classified in the “diverse classes” section, few reports exist in the literature regarding interactions with the HIV targets discussed here, except for vitamin D derivatives and dinoprostone, which require further robust studies to elucidate their mechanisms of action.

Regarding the toxicity profiles of the various compounds, latanoprost, cilastatin, dinoprostone, pioglitazone, and cefprozil demonstrated the most favorable outcomes across the nine evaluated parameters. Conivaptan and droperidol exhibited medium to high toxicity profiles, particularly concerning genotoxicity, with results approaching the maximum limit. Calcitriol and ziprasidone were classified as having high toxicity across most parameters.

Although all compounds evaluated are FDA-approved, adverse effects remain an important consideration, especially in PLHIV, who require lifelong combination therapy. Therefore, toxicity assessment is critical when considering drug repositioning, particularly with respect to potential interactions with existing HAART regimens, as monotherapy is not recommended.

None of the compounds violated Lipinski’s rule of five. However, violations were more frequent under the more restrictive GSK rule, particularly among icosapent ethyl, calcitriol, ziprasidone, and ezetimibe, which also violated the Pfizer rule. Since the GSK rule imposes stricter limits on molecular weight and lipophilicity, such violations may affect bioavailability and tissue distribution. Additionally, most compounds violating the Pfizer rule were also classified as highly toxic, reinforcing the importance of integrating drug-likeness and toxicity metrics in future drug repositioning strategies.

QSAR models and molecular docking assays evaluate potential interactions between molecules and target proteins using distinct methodologies. This fundamental difference explains why many interactions predicted with high probability by QSAR models were not corroborated by molecular docking assays.^65,66^ QSAR relies on molecular descriptors that capture the topological and physiochemical properties of the analyzed compounds, with the correlation between the descriptors and the expected experimental activity modeled through statistical or machine learning models. However, it is well recognized that QSAR models are highly dependent on the nature of their training set. They tend to exhibit strong predictive performance when applied to compounds that are structurally related to those in the training set, but their predictive power decreases when they are used to evaluate structurally diverse molecules, as is the case in the present study.^67^

In contrast, molecular docking does not require prior ligand activity data but depends on accurate structural information on the target protein. Although docking scoring functions often show limited quantitative correlation with experimental binding affinities, they are generally effective in discriminating between active and inactive compounds by estimating relative binding energies rather than absolute interaction energies.^67^ Taken together, these findings underscore the complementary nature of ligand-based and structure-based approaches and support their combined application in virtual screening pipelines.

These discrepancies between the results obtained from QSAR models and molecular docking highlight the need for caution when interpreting computational findings. It is important to recognize that virtual screening outcomes are directly affected by errors inherent in the input data, which propagate through the constructed models and consequently influence their predictions. Nonetheless, in high-throughput screening assays, the expected hit rate is typically below 1%, which supports the use of computational approaches as an alternative to enhance predictive success rates.^68^ Additionally, approximately 39% of the molecules evaluated in this study were confirmed by the PASS Online software^69^ as having potential activity against HIV-1, HIV-2, or exhibiting general antiviral properties.

By employing virtual screening, some molecules identified as promising hits may be commercially unavailable, either because they have been discontinued by the pharmaceutical industry, withdrawn from the market due to regulatory issues, or retained only for highly specific applications. This does not appear to be the case for the hits identified in the present study, as all molecules are commercially available, and because we chose to use a library of molecules approved by the USA regulatory agency (FDA).

Drug repurposing substantially reduces the risks and costs associated with drug development, as key stages such as preclinical evaluation, toxicological testing, chemical optimization, and formulation have already been completed. As a result, the development timeline can be reduced from approximately 15 years to about 6.5 years, with estimated costs decreasing from 2 to 3 billion to around 300 million United States dollars.^70^ In line with this, repurposed drugs exhibit a lower overall probability of failure compared to de novo drug discovery approaches.^71^

Despite these advantages, drug repositioning faces important challenges, particularly when compounds remain under patent protection, which limits access to safety and efficacy data. Similar constraints apply to discontinued or abandoned drug candidates that progressed through early clinical development but failed to achieve regulatory approval due to insufficient efficacy, safety concerns, or financial limitations. Given that nearly 90% of drug candidates entering clinical trials do not reach approval, restricted data access often makes repositioning efforts more feasible for patent-holding pharmaceutical companies than for academic or small biotechnology groups.^71^

## Conclusions

Regarding the drugs evaluated, it is important to emphasize that these results represent preliminary findings. Nonetheless, this study, combined with existing literature, highlights pazopanib, panobinostat, fluvastatin, and calcitriol as promising candidates in this virtual screening approach. Further research is essential to explore and validate these molecular interactions and assess their clinical relevance. The QSAR models developed alongside molecular docking assays have provided valuable insights into potential novel compounds for HIV treatment. Based on these models, a software tool has been created to facilitate the academic community’s screening of additional molecules.

Utilizing a computational approach to prioritize molecules, particularly those with multitarget potential, significantly accelerates the drug discovery process for complex diseases like HIV. However, this also highlights the critical need for robust experimental studies to validate the predictive findings and confirm the efficacy of the identified compounds.

## Materials and Methods

### Data Search and Selection

The study began with the search and selection of data from high-throughput screening (HTS) studies available in the PubChem database (https://pubchem.ncbi.nlm.nih.gov/) to train and validate QSAR models. PubChem is an open-access chemical database that provides extensive information on chemical structures, biological activities, safety, and toxicity. The “bioassays” entry was used for data search, using “HIV” as the keyword and focusing on studies aimed at developing new HIV drugs. Only bioassays containing at least 500 active molecules were included to ensure a sufficient data set for both model training and validation.

The BAMBU tool was used to train and validate the QSAR models. BAMBU is a command-line software that autogenerates QSAR models using HTS data as input.^72^ Bioassay data were downloaded from PubChem via the *bambu-download* command, which generates a CSV file containing molecular information. Each molecule is represented by its InChl strings in the “InChl” column and labeled as either “active” or “inactive” in the “activity” column.

Table 4 summarizes the selected studies, listing the AID (PubChem assay identification number), study targets, total number of compounds tested, and the distribution between active and inactive compounds. While most studies focused on discovering new HIV drugs, some targeted glycoprotein gp160 and sialate acetyl esterase, aiming to identify compounds relevant for vaccine development. These targets were also included due to their biological significance and the potential value of the compound libraries. Based on the established criteria, 14 studies were selected for QSAR model training and validation.

**Table 4:** HTS Studies for the Development of New Drugs or Vaccines Selected from the PubChem Database, as Well as Their Targets, Number of Substances Tested and the Division between Active and Inactive^a^

AID	study target	tested substances	active substances	inactive substances
651571	surface glycoprotein gp160	1.474	1.328	146
565	reverse transcriptase	65.239	1.250	63.989
704	Rev protein	96.889	769	96.120
1117361	Tat protein	3.634	904	2.643
624416	surface glycoprotein gp160	362.877	1.546	361.331
651604	surface glycoprotein gp160	1.474	1.402	72
624170	GLS protein	409.400	846	401.810
1053197	sialate acetylesterase	370.256	2.555	367.701
1117319	Vif protein	262.345	1.501	248.801
743269	lens epithelial-derived growth factor p75 and integrase	370.276	2.355	367.921
1986	envelope glycoprotein (*gp41)	292.483	891	291.592
1053136	lens epithelial-derived growth factor p75 and integrase	2.020	731	1.289
463187	Nef protein	220.335	1.495	218.840
372	ribonuclease H	99.840	770	98.999

a Source: https://pubchem.ncbi.nlm.nih.gov/ (bioassays).

### Training and Validation of QSAR Models

For model development, the data were initially downloaded and preprocessed using the *bambu-preprocess* command. At this stage, the raw data set was partitioned into two subsets: 75% for training and 25% for validation. Molecules, originally in InChI format, were subsequently converted into different representations. BAMBU uses the MORDRED package to compute over 1200 structural and physicochemical molecular descriptors (both 2D and 3D). Additionally, models were generated employing Morgan fingerprints (1024 or 2048 bits) and vector representations via Mol2Vec. To mitigate the significant class imbalance in the data set, a random subsampling technique was applied. This process utilized the following packages: Pandas (https://pandas.pydata.org/), NumPy (https://numpy.org/), Scikit-learn (https://scikit-learn.org) and Imbalanced-learn (https://imbalanced-learn.org/).

Following preprocessing, the models were trained using the *bambu-train* command. This training process utilized the subset of training data to identify the best classification model through the application of optimized hyperparameters. BAMBU incorporates fast and lightweight automated machine learning (FLAML), a machine learning framework developed by Microsoft, which automatically selects the most suitable model and hyperparameters for each study. FLAML’s methodology includes algorithms such as random forest, gradient boosting, logistic regression, and extra-trees.

Once the optimal hyperparameter configuration was selected, the predictive models were refined using the training data. The *bambu-validate* command was employed to perform validation, calculating accuracy, recall, precision, F1 score, and the area under the ROC curve (AUC), with AUC serving as the primary evaluation metric. Based on the classification metrics derived from both the training and test sets, *p-values* were calculated for each metric. An additional step to evaluate the selected model involved *k-fold* cross-validation (*k* = 10) on the training data set. In this process, the data set was partitioned into *k* partitions, and in each iteration, *k – 1* partitions were used for training, while the remaining subset was used for validation. Figure 1 illustrates the BAMBU workflow in detail.

**Figure 1:**
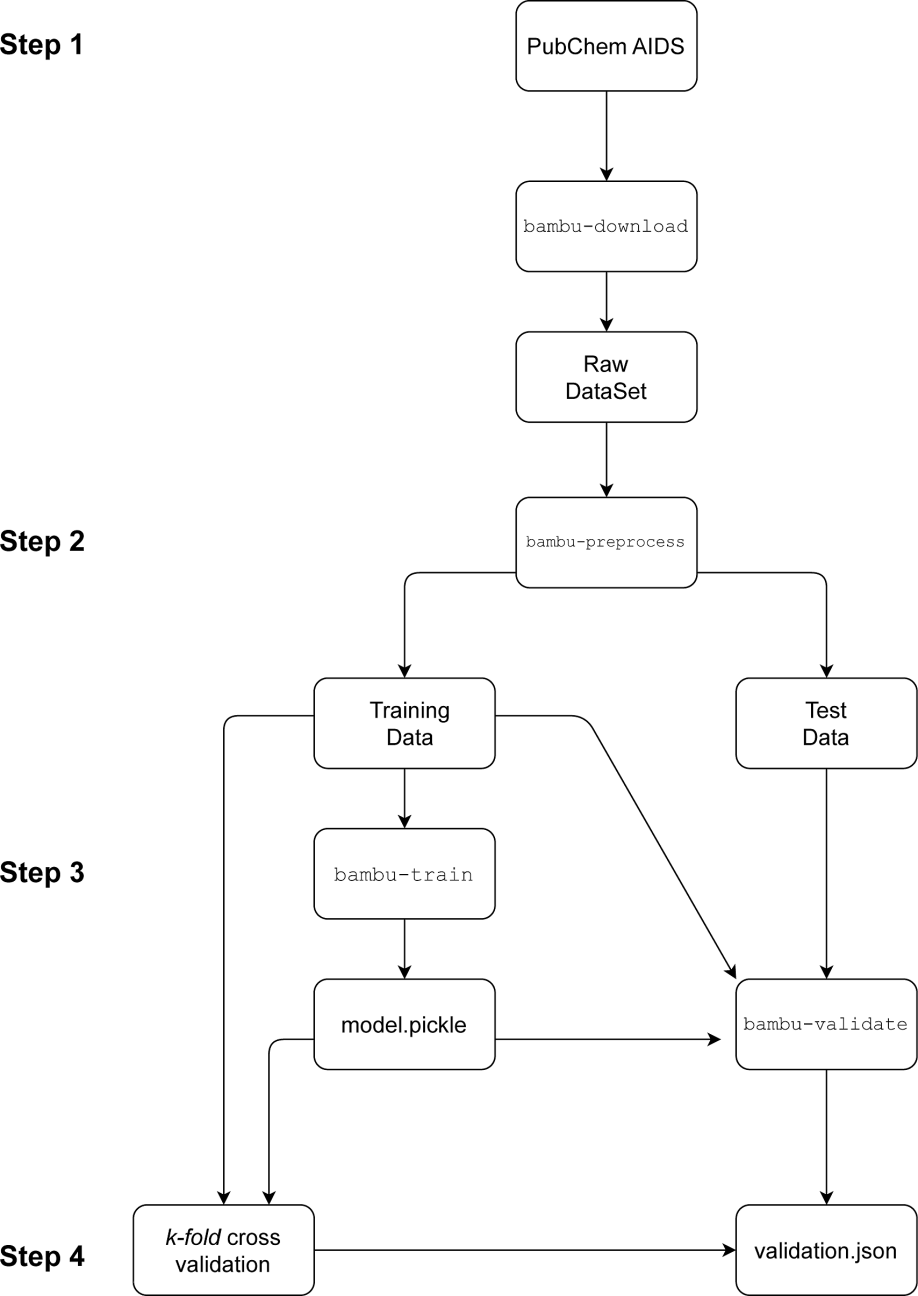
Flowchart of steps for training and validating QSAR models using BAMBU.

### Virtual Screening

Virtual screening encompasses the ligand-based design or repositioning step. For this process, the best QSAR models developed for each target were employed. Molecules from the Zinc15 database (https://zinc15.docking.org/), specifically from the “*FDA Approved*” library (comprising 1.615 drugs), underwent an initial filtering. Subsequently, an average score was computed for these molecules based on the results generated by the different models. The top 1.000 promising molecules were then selected for the molecular docking phase.

### Molecular Docking

In the molecular docking phase, simulations of docking between proteins and small ligands were performed using AutoDock Vina,^73^ which employs its standard empirical scoring function, based on ligand–receptor interaction energy terms. No custom modifications were made to the weights or individual components of the scoring function. Ligand and receptor structures were prepared using AutoDock Tools.^74^ HIV target structures were obtained from the Protein Data Bank (PDB), and relevant residues for each target were identified through a comprehensive literature review, including prior studies detailing their mechanisms of action or molecular docking investigations. When critical residues were not found in the literature, the active site predictor PrankWeb^75^ was employed. Additionally, the protease structure was incorporated in the docking process due to its critical role in HIV, despite the absence of a generated QSAR model, as no HTS data were available for this structure in PubChem. The identified residues are summarized in Table 2, available in the Supporting Information.

For each target structure, a grid box of 10 Å in all three axes was generated around the selected residues. The docking results were subsequently processed using the PLIP tool,^76^ which calculated binding interaction scores for each protein–ligand complex. These scores were derived from the percentage of relevant residues, identified in the literature review, that exhibited predicted interactions with a specific ligand.

Protein–ligand interaction fingerprints (PLIFs) were then utilized to compute an average score across all targets, enabling the ranking of molecules. High-affinity targets were defined as those exhibiting binding energies ≥−7.0 kcal/mol and possessing at least 0.3 heavy atoms in the ligand.^77,78^ Only molecules with at least one high-affinity target were selected for the subsequent phase.

### Analysis of Pharmacokinetic Characteristics, Toxicity, and Promiscuous Compounds

The pharmacokinetic and toxicological properties, alongside structural alerts for molecular promiscuity, were evaluated for each molecule using the ADMETLab 3.0 web server^79^ Following these comprehensive analyses, a final list of the 20 most promising molecules from the analyzed library was compiled.

## Supplementary Material



## Notes

Table SA—list of the 100 molecules from the FDA-approved compound library selected for molecular docking; and Table SB—top 20 molecules from the FDA-approved drug library with high-affinity targets identified by molecular docking for different HIV-related targets. Binding energy and binding efficiency are reported in kcal/mol. The protein–ligand interaction fingerprint was compared with residues previously described in the literature or predicted using specific tools, which were considered relevant for the activity of molecules targeting the proteins (PDF)
