# Supplementary material for: Virtual Screening of FDA-Approved Compounds: Exploring New Alternatives for HIV Treatment
Source: ACS Omega. 2026 Feb 18;11(8):13062–73. doi: 10.1021/acsomega.5c06562 (PMC12961502; doi:10.1021/acsomega.5c06562)
Supplement: Supplementary file 1 [file ao5c06562_si_001.pdf]

Virtual Screening of FDA-Approved Compounds: Exploring New Alternatives for HIV Treatment

*Daniela P. Martinez; Frederico S. Kremer**

Technological Development Center, Federal University of Pelotas, Pelotas, Rio Grande do Sul, Brazil.

Table A – List of the 100 molecules from the FDA-approved compound library selected for molecular docking.

A	B	C	D	E	F	G	H	I	J
ZINC000007<br>997966	ZINC000001<br>530968	ZINC000003<br>830999	ZINC000100<br>036924	ZINC000000<br>009073	ZINC000001<br>481815	ZINC000003<br>995807	ZINC000084<br>843283	ZINC000003<br>995809	ZINC000000<br>001411
ZINC000252<br>679615	ZINC000000<br>001688	ZINC000000<br>000922	ZINC000035<br>342789	ZINC000000<br>057624	ZINC000003<br>830391	ZINC000013<br>550868	ZINC000169<br>621228	ZINC000000<br>008492	ZINC000003<br>940470
ZINC000169<br>289767	ZINC000034<br>220093	ZINC000004<br>474443	ZINC000022<br>116608	ZINC000004<br>475353	ZINC000000<br>057278	ZINC000001<br>554274	ZINC000019<br>168887	ZINC000000<br>002028	ZINC000004<br>215234
ZINC000005<br>733652	ZINC000003<br>794794	ZINC000003<br>875368	ZINC000095<br>564694	ZINC000000<br>034157	ZINC000084<br>441937	ZINC000003<br>978006	ZINC000084<br>589076	ZINC000000<br>538275	ZINC000003<br>872055
ZINC000003<br>812865	ZINC000001<br>482049	ZINC000003<br>776970	ZINC000022<br>116612	ZINC000003<br>938704	ZINC000014<br>879992	ZINC000000<br>006226	ZINC000000<br>035804	ZINC000003<br>989268	ZINC000253<br>387843
ZINC000003<br>831490	ZINC000003<br>860453	ZINC000004<br>098633	ZINC000000<br>602632	ZINC000003<br>918087	ZINC000016<br>052277	ZINC000100<br>006264	ZINC000030<br>691797	ZINC000003<br>927198	ZINC000003<br>827556
ZINC000002<br>568036	ZINC000000<br>895199	ZINC000000<br>001554	ZINC000003<br>920266	ZINC000003<br>916214	ZINC000000<br>002216	ZINC000003<br>871967	ZINC000000<br>002055	ZINC000004<br>468778	ZINC000006<br>382803
ZINC000035<br>342787	ZINC000007<br>997568	ZINC000003<br>830215	ZINC000006<br>409735	ZINC000003<br>830993	ZINC000003<br>786192	ZINC000169<br>621223	ZINC000001<br>690324	ZINC000005<br>133378	ZINC000000<br>057255
ZINC000002<br>015035	ZINC000000<br>020243	ZINC000000<br>020255	ZINC000011<br>679756	ZINC000012<br>414057	ZINC000003<br>995811	ZINC000169<br>621200	ZINC000068<br>153186	ZINC000000<br>518554	ZINC000001<br>530788
ZINC000003<br>952881	ZINC000000<br>033882	ZINC000003<br>917708	ZINC000001<br>883067	ZINC000000<br>003911	ZINC000052<br>509463	ZINC000096<br>006023	ZINC000003<br>926298	ZINC000000<br>896755	ZINC000000<br>001758

Column A – Molecules 1–10; Column B – Molecules 11–20; Column C – Molecules 21–30; Column D – Molecules 31–40; Column E – Molecules 41–50; Column F – Molecules 51–60; Column G – Molecules 61–70; Column H – Molecules 71–80; Column I – Molecules 81–90; and Column J – Molecules 91–100.

Table B – Top 20 molecules from the FDA-approved drug library with high-affinity targets identified by molecular docking for different HIV-related targets. Binding energy and binding efficiency are reported in kcal/mol. The protein-ligand interaction fingerprint was compared with residues previously described in the literature or predicted using specific tools, which were considered relevant for the activity of molecules targeting the proteins.

**ZINC ID**	**DrugBank ID**	**Name**	**High Affinity Target**	**Target**	**Interactions**	**Binding Energy**	**Ligand Efficiency**	**Score PLIF**
ZINC000011617039	DB06589	Pazopanib	5ivq	Protease	Asp29(A), Asp30(A), Gly27(A), Ile50(B), Val82(A).	-9.9	-0.32	35.28%
ZINC000012503187	DB00872	Conivaptan	5ivq	Protease	Val32 (A), Ala28 (A), Ile47 (A), Asp30 (B), Val82 (B), Val32 (B), Gly48 (A), Ile50 (A), Pro81 (B), Ile47 (B), Asp29 (B), Gly48 (B).	-11.6	-0.31	34.84%

ZINC000035801098	DB05039	Indacaterol	3lp0	Reverse Transcriptase	Tyr181 (A), Leu234 (A), Val106 (A), Pro95 (A), Tyr188 (A), His235 (A), Leu100 (A), Trp229 (A).	-8.8	-0.30	34%
			5ivq	Protease	Ala28 (A), Ile47 (A), Asp30 (B), Asp25 (B), Arg8 (A), Ala28 (B), Ile50 (A), Ile50 (B), Asp29 (B).	-9.4	-0.32	
ZINC000001886617	DB01095	Fluvastatin	3lp0	Reverse Transcriptase	Asn103 (A), Tyr181 (A), Leu234 (A), Glu138 (B), Val106 (A), Tyr318 (A), Pro95 (A),	-10.7	-0.36	33.83%

					Tyr188 (A), Val179 (A), Leu100 (A), Trp229 (A).			
ZINC000012468792	DB00654	Latanoprost	3lp0	Reverse Transcriptase	Asn103 (A), Tyr181 (A), Leu234 (A), Glu138 (B), Val106 (A), Lys101 (A), Pro95 (A), Tyr188 (A), Val179 (A), Phe227 (A), Leu100 (A), Trp229 (A).	-9.3	-0.30	32.47%
ZINC000003785276	DB0887	Icosapent Ethyl	3f9k	Integrase	Lys14 (A), Leu363 (C), Gln168 (A), Lys402 (C), Trp131 (B), Glu10 (A), Lys360 (C), Ile403 (C),	-7.4	-0.31	32.43%

					Phe406 (C), Thr399 (C).			
			3lp0	Reverse Transcriptase	Asn103 (A), Tyr181 (A), Leu234 (A), Tyr318 (A), Val106 (A), Pro95 (A), Tyr188 (A), Val179 (A), Phe227 (A), Leu100 (A), Trp229 (A).	-8.3	-0.35	
ZINC000000897256	DB00983	Formoterol	3lp0	Reverse Transcriptase	Tyr181 (A), Leu234 (A), Val106 (A), Tyr188 (A), Val179 (A), Leu100 (A), Trp229 (A).	-8.6	-0.34	32.13%
ZINC000002599970	DB00983	Formoterol	3lp0	Reverse Transcriptase	Asn103 (A), Tyr181 (A), Leu234 (A), Glu138 (B),	-9.2	-0.37	32%

					Val106 (A), Tyr318 (A), Pro95 (A), Tyr188 (A), Leu100 (A), Trp229 (A).			
			5ivq	Protease	Val32 (A), Ala28 (A), Asp30 (B), Asp25 (A), Asp25 (B), Ala28 (B), Ile50 (B), Ile84 (A), Asp29 (B), Gly27 (B).	-7.9	-0.32	
ZINC000100015048	DB00136	Calcitriol	5ivq	Protease	Val32 (A), Ala28 (A), Ile47 (A), Asp29 (A), Asp30 (B), Asp30 (A), Ala28 (B), Ile50 (A),	-10.2	-0.34	31.91%

					Ile50 (B), Ile84 (B), Ile84 (A), Asp29 (B).			
ZINC000019796080	DB00450	Droperidol	3f9k	Integrase	Glu13 (A), Leu363 (C), Gln168 (A), Lys402 (C), Trp131 (B), Lys360 (C), Ile403 (C), Phe406 (C).	-8.6	-0.31	31.89%
			3lp0	Reverse Transcriptase	Pro236 (A), Asn103 (A), Tyr181 (A), Leu234 (A), Val106 (A), Pro95 (A), Tyr188 (A), Val179 (A), Phe227 (A), Leu100 (A), Trp229 (A).	-11.1	-0.40	

			5ivq	Protease	Ala28 (A), Val32 (B), Asp25 (B), Ala28 (B), Ile50 (A), Ile50 (B)	-9.2	-0.33	
			5uqe	GLS Protein	Asn335 (D), Val334 (D), Lys481 (D), Pro313 (D), Cys463 (D), Lys289 (D), Tyr466 (D), Val484 (D), Ser286 (D).	-8.7	-0.31	
ZINC000000538550	DB00246	Ziprasidone	5ivq	Protease	Val32 (A), Ile47 (A), Asp30 (B), Val32 (B), Asp30 (A), Asp25 (A), Asp25 (B), Ala28 (B), Ile50 (A),	-9.9	-0.35	31,76%

					Ile47 (B), Asp29 (B).			
			5uqe	GLS Protein	Asn335 (D), Tyr249 (D), Lys289 (D), Asp248 (D), Glu381 (D), Leu505 (D), Lys245 (D), Tyr414 (D).	-8.7	-0.31	
ZINC000003819392	DB08931	Riociguat	5uqe	GLS Protein	Asn335 (D), Val334 (D), Lys289 (D), Asp467 (D), Tyr466 (D), Asn388 (D), Ser286 (D)	-9.8	-0.32	31.59%
ZINC000000403011	DB00598	Labetalol	3lp0	Reverse Trasnscriptase	Asn103 (A), Tyr181 (A), Leu234 (A), Val106 (A), Tyr188 (A), Val179 (A), Phe227 (A),	-9.5	-0.40	31.56%

			Leu100 (A), Trp229 (A).		
	5ivq	Protease	Val32 (A), Ala28 (A), Ile47 (A), Asp30 (B), Val32 (B), Ala28 (B), Ile50 (A), Ile50 (B), Ile84 (A), Asp29 (B).	-8.3	-0.35
	5uqe	GLS Protein	Asn335 (D), Val334 (D), Asn319 (D), Gly315 (D), Tyr466 (D), Asn331 (D), Ser469 (D).	-7.5	-0.31
	6b72	Nef Protein	Phe90 (B), His116 (A), Ile87 (B), Gln73 (A), Arg71 (A),	-7.3	-0.30

					Trp113 (B), Val70 (A), Thr117 (B).			
ZINC000004095696	DB01597	Cilastatin	3lp0	Reverse Transcriptase	Leu234 (A), Glu138 (B), Val106 (A), Pro95 (A), Tyr188 (A), Val179 (A), Leu100 (A), Trp229 (A).	-7.8	-0.32	31.34%
			5ivq	Protease	Ala28 (A), Ile47 (A), Val82 (B), Asp29 (A), Arg8 (B), Gly48 (A), Gly27 (A), Ile50 (B), Ile50 (A), Pro81 (B), Ile84 (B), Ile84 (A), Gly27 (B).	-7.3	-0.30	

ZINC000051951647	DB06603	Panobinostat	3lp0	Reverse Transcriptase	Tyr181 (A), Leu234 (A), Tyr318 (A), Val106 (A), Pro95 (A), Tyr188 (A), Val179 (A), His235 (A), Phe227 (A), Leu100 (A), Trp229 (A).	-10.1	-0.39	31.20%
			5ivq	Protease	Ala28 (A), Asp30 (B), Ile47 (A), Asp29 (A), Val32 (B), Asp30 (A), Asp25 (A), Ala28 (B), Ile50 (A), Ile50 (B), Ile84 (A).	-9.5	-0.37	
ZINC000003810860	DB00973	Ezetimibe	3lp0	Reverse Transcriptase	Asn103 (A), Tyr181 (A),	-10.5	-0.35	31.11%

					Leu234 (A), Glu138 (B), Val106 (A), Lys101 (A), Pro95 (A), Tyr188 (A), Val179 (A), Phe227 (A), Leu100 (A), Trp229 (A).			
			5ivq	Protease	Val32 (A), Ala28 (A), Val82 (B), Val32 (B), Thr80 (B), Ala28 (B), Ile50 (A), Ile50 (B), Ile84 (A).	-9.7	-0.32	
ZINC000003830713	DB00917	Dinoprostone	3lp0	Reverse Transcriptase	Asn103 (A), Tyr181 (A), Leu234 (A), Lys101 (A), Val106 (A),	-8.0	-0.32	31.06%

					Tyr188 (A), Val179 (A), His235 (A), Leu100 (A), Trp229 (A).			
ZINC000000968326	DB01132	Pioglitazone	3f9k	Integrase	Glu13 (A), Lys14 (A), Leu363 (C), Trp131 (B), Glu10 (A), Lys402 (C), Ile403 (C), Phe406 (C).	-7.7	-0.31	30.87%
			3lp0	Reverse Transcriptase	Asn103 (A), Tyr181 (A), Leu234 (A), Glu138 (B), Val106 (A), Pro95 (A), Tyr188 (A), Val179 (A), His235 (A), Leu100 (A), Trp229 (A).	-8.9	-0.36	

			5ivq	Protease	Ala28 (A), Val82 (B), Gly27 (A), Pro81 (B), Ile50 (A), Ile50 (B), Ile84 (A), Gly48 (B).	-8.1	-0.32	
			5uqe	GLS Protein	Gly315 (D), Tyr466 (D), Val484 (D), Ser314 (D), Ser286 (D), Ser469 (D).	-7.9	-0.32	
			6b72	Nef Protein	Phe90 (B), Tyr120 (B), His116 (A), Gln73 (A), Trp113 (B), Val70 (A), Thr117 (B), Asp86 (B).	-7.6	-0.30	
ZINC000004474443	DB01150	Cefeprozil	5ivq	Protease	Asp29 (A), Ile47 (A),	-8.7	-0.32	30.86%

					Asp30 (B), Gly48 (A), Gly27 (A), Ile50 (B), Ile50 (A), Asp29 (B), Gly48 (B).			
ZINC000022010649	DB06603	Panobinostat	3f9k	Integrase	Glu13 (A), Lys14 (A), Glu167 (A), Leu363 (C), Trp131 (B), Glu10 (A), Lys402 (C), Ile403 (C), Phe406 (C)	-8.2	-0.32	30.67%
			3lp0	Reverse Transcriptase	3lp0': 'Pro236 (A), Leu234 (A), Glu138 (B), Val106 (A), Tyr188 (A), Val179 (A), Phe227	-9.7	-0.37	

			(A), Leu100 (A)		
	5ivq	Protease	Val32 (A), Ala28 (A), Ile47 (A), Val82 (B), Asp29 (A), Asp30 (A), Asp25 (A), Ile50 (B), Ile50 (A), Ile84 (B), Ile84 (A), Leu23 (B)	-8.8	-0.34
